# Screening and health education practices related to cardiovascular risk factors among adults: results of a households survey in Sousse, Tunisia

**DOI:** 10.1038/s41598-022-23600-3

**Published:** 2022-11-05

**Authors:** Nawel Zammit, Waad Ben Belgacem, Rim Ghammam, Sihem Ben Fredj, Rim Krifa, Mohamed Ouertani, Amani Maatouk, Jihene Maatoug, Hassen Ghannem

**Affiliations:** 1 Department of Epidemiology (LR19SP03), Faculty of Medicine of Sousse, Farhat Hached University Hospital, University of Sousse, 4000 Sousse, Tunisia; 2 Department of Epidemiology, Farhat Hached University Hospital, 4000 Sousse, Tunisia

**Keywords:** Cardiology, Health care, Risk factors

## Abstract

In Tunisia, despite the implementation of national strategies to prevent some of cardiovascular risk factors, these diseases still represent the leading cause of death. The current study aimed to determine the prevalence of cardiovascular risk factors and assess the screening and health education practices related to these factors among the adults of Sousse (Tunisia). A cross-sectional study was conducted in 1000 households in Sousse, Tunisia. To collect data, a team of trained medical doctors administered by interview a pre-tested questionnaire and performed blood pressure and anthropometric measures at the participants’ homes. In total, 1977 adults participated in the study. Their mean age was 39.8 (± 13.8) years. The Females/Males sex ratio was 1.5. Weight excess and lack of physical activity were found in 65.4% and 60.7% of participants. Screening for obesity and android obesity were reported by 36.6% and 5.7% of health services users. Advices from a healthcare professional to practice the recommended physical activity and eat healthily were reported by less than a third of these latter. Screening and health education practices related to cardiovascular risk factors should be reinforced in the Tunisian healthcare facilities. The implementation of multisectoral actions is necessary at the national level in order to obtain an environment that promotes a healthy lifestyle.

## Introduction

Each year, cardiovascular diseases (CVD) kill 17.9 million people^1^. In 2015, they were responsible for 31% of all deaths worldwide and 50% of premature deaths (before the age of 70 years) by a non-communicable disease (NCD)^2,3^. Over 80% of CVD-related deaths occur in low and middle-income countries, especially premature deaths^1,3,4^. The genesis of CVD is multifactorial. Behavioral risk factors (RFs) including tobacco use, excessive alcohol consumption, lack of physical activity and unhealthy diet are modifiable^5–7^. The control of metabolic ones such as obesity, dyslipidemia, and diabetes as well as high blood pressure, can also reduce the cardiovascular risk^5–7^. In fact, the compliance with a healthy lifestyle and the medicinal treatment of these RFs makes it possible to avoid 80% of premature ischemic attacks^8^ while early detection and treatment of CVD can prevent almost 40% of their related deaths^9,10^.

In Tunisia, the implementation of a national strategy for the management of hypertension and diabetes since 1999 and another strategy to reduce tobacco use since 2008 were not sufficient to decrease CVD mortality in a sustained manner^11,12^. Indeed, in 2013, 2015 and 2017, CVD caused 29%, 24.5% and 26% of all deaths respectively^11,12^. In fact these strategies were rarely revised^13^. Furthermore, the last evaluation of these strategies conducted between 2015 and 2017 focused mainly on the planning aspect^13^. Evaluating the screening and health education practices related to CVD RFs in the Tunisian healthcare facilities is therefore necessary to guide the implementation of future CVD prevention actions in Tunisia.

Objective: To determine the prevalence of CVD RFs and assess the practices of screening and health education related to these factors among the adults in Sousse, Tunisia.

## Materials and methods

### Type of study

A cross-sectional study was conducted in the governorate of Sousse in 2014 among adults aged over 18 years.

### Study population

Given that the prevalences of the different CVD RFs are comprised between 8.4% (for dyslipidemia^14^) and 32%, (for smoking^15^) and considering a precision of 3%, a type one error of 5%, and a cluster effect of 2, a total of at least 1856 participants was required. To recruit participants, a two-stages cluster sampling was performed to select 1000 households from the governorate of Sousse. Initially, to reduce the geographic dispersion of the sample and the cost of the survey, three from the 15 delegations of the governorate of Sousse were randomly selected. They were the delegations of Sousse-Jawhara, Sousse-Riadh, and Msaken. Then, 16 districts were randomly selected from these three delegations proportionally to their population densities. During data collection, all adults aged over 18 years that were present in the households of these districts and who gave informed consent to participate were included in the study.

### Data collection

A questionnaire, established in line with the objectives of the study and written in Arabic was tested among a convenience sample of 110 participants that were not included later to the study sample. Cultural acceptability and the understandability of the items were assessed. Unclear items and those that were difficult to understand by two or more participants were reformulated considering their comments and the opinion of experts (Three public health specialist and one family medicine doctor). The final version of the questionnaire was administered by interview to participants in their homes by trained physicians. The collected data included socio-demographic characteristics, medical history, dietary and physical activity habits, tobacco use, alcohol consumption, healthcare utilization, screening and health education practices related to the CVD RFs in the visited healthcare facilities during the previous year. After administration of the questionnaire, the physicians measured weight, height, waist circumference, systolic and diastolic blood pressures for each participant. Anthropometric measurements were performed in bare feet with light clothing. Body weight was recorded to the nearest 0.1 kg using portable electronic scales (PS 07 from Beurer). Standing height was recorded to the nearest 0.5 cm. Waist circumference was measured with a tape measure at the midpoint between the last palpable rib and the iliac crest. Blood pressures were recorded twice for each participant after a 15-min of rest using electronic sphygmomanometers (Omron M3 Intellisense (HEM-7051-E)).

### Variables definition

- Socioeconomic level was determined according to a socioeconomic index calculated from the following variables: access to electricity, access to drinking water, access to a telephone, number of cars, number of refrigerators, number of air conditioners, number of television sets, number of computers connected to the internet, number of toilets, and number of washing machines. In order to determine the socio-economic class according to the socio-economic level score, factor analysis and a hierarchical analysis were performed. Accordingly, 4 classes of socio-economic level were obtained: low, low to medium, medium to high, and high. Finally, a grouping of the categories "low to medium" and "medium to high" was performed to obtain the "medium" category.
- The lines of the visited healthcare facilities: The first line included the primary health care centers or the medical office of family doctors. The second line included the regional hospitals or the medical office of specialised doctors. The third line included the university hospitals or the private clinics.
- The sector of the visited healthcare facility was either public or private. Public facilities were those financed by the Tunisian health ministry (primary health care centers, regional and university hospitals). Private facilities were those funded by individuals (medical offices of doctors and clinics).
- History of hypercholesterolemia, diabetes, high blood pressure, and cardiovascular disease were self reported.
- The current tobacco use was assessed using the following question: "Currently, do you use any type of tobacco products?" Those who answered by "yes" were considered as current tobacco users.
- The practice of physical activity as recommended by the World Health Organization (WHO)^16^ was assessed via the following question: "Do you currently practice 30 min or more of physical activity for 5 days a week?". Those who answered by "yes" to this question were considered to be in compliance with this recommendation.
- The consumption of fruits and vegetables as recommended by the WHO^17^ was assessed via the following question: "Do you currently eat 5 servings of fruits and vegetables per day? Those who answered by "yes" to this question were considered to be in compliance with this recommendation.
- Assessment of cardiovascular RFs in the visited healthcare facilities during the previous year was measured via the following question: "If you have visited a healthcare facility during the previous year, did a health professional evaluate your smoking status, weight, waist circumference, cholesterolemia, glycaemia or blood pressure?" Possible responses for each measure were "yes," "no," or "I'm not sure."
- Information about its global CVD risk in the visited healthcare facilities was assessed via the following question: "If you have visited a healthcare facility during the previous year, did a health professional inform you about the level of your CVD risk estimated on the basis of your individual RFs?” Possible responses for each measure were "yes," "no," or "I'm not sure."
- Health education practices related to the CVD RFs in the visited healthcare facilities during the previous year were assessed via the following question: "If you have visited a healthcare facility during the previous year, did a health professional advise you on how to eat healthily, to engage in more physical activity or to quiet tobacco if you are a tobacco user?” Possible responses for each item were "yes," "no," or "I'm not sure."
- History of cardiovascular disease was assessed using the following question: “Have you been diagnosed with or have you been treated for a heart attack or a stroke?”. Possible responses for each item were "yes" or "no".
- The blood pressure was considered elevated if the average systolic blood pressure was ≥ 140 mmHg and/or the average diastolic blood pressure was ≥ 90 mmHg after two measures following a 15 min of rest^18^.
- Body mass index (BMI) was calculated as the patient’s weight in kg divided by height in m^2^. Overweight was defined as BMI of 25 to 29.9, and obesity was defined as BMI ≥ 30^19^.
- Android obesity was determined according to the International Diabetes Federation (IDF) definition by a waist circumference ≥ 94 cm for men and ≥ 80 cm for women^20^.

### Data analysis

Data entry and analysis were performed using SPSS (Statistical Package for Social Sciences) version 11.0 software. Descriptive statistics were reported as frequencies for categorical variables and as means and standard deviations for quantitative ones. The Chi^2^ test was used to compare percentages. The responses: "I'm not sure” were considered as missing values and were excluded from cross comparisons. All statistical tests were 2-tailed, and *p* values < 0.05 were considered statistically significant.

### Ethical considerations

The current study was carried out in accordance with the ethical principles of the Declaration of Helsinki. It was approved by the Ethical Committee of Farhat Hached University Hospital (Institutional review board code: 00008937). The agreement of the Ministry of Health and the Governor of Sousse were obtained before the beginning of the study. Participation was voluntary. Informed consent was obtained from each participant. Explanations on the purpose of the study, anonymity and confidentiality of collected data were provided by the investigating physicians beforehand. Participants who required healthcare during data collection were referred to the appropriate healthcare facilities.

### Ethical approval

The current study was carried out in accordance with the ethical principles of the Declaration of Helsinki. The procedures of the study were approved by the Research Ethics Committee of University Hospital Farhat Hached (Institutional review board code: 00008937). 

### Consent to participate

Informed consent was obtained from all individual participants included in the study.

## Results

In total, 1977 adults accepted to participate in the study. Their age ranged from 18 to 79 years with an average of 39.8 (± 13.8) years. Females accounted for 1200 (61%). Regarding the socioeconomic level, a low level was found in 144 (7.3%) participants, a medium level was found in 1633 (83%) participants and a high level was found in 196 (9.9%) participants. Otherwise, lack of social security coverage was reported by 401 (20.3%) participants. The 1st, the 2nd and the 3rd lines of care were used by 514 (26%), 489 (24.7%) and 456 (24.6%) of them (*p* = 0.139). Private facilities were more visited by 681 (34.4%) participants, public facilities were more visited by 503 (25.4%) participants while 154 (7.8%) were using both sectors.

Tobacco use and alcohol consumption were reported by 47.7% and 15.5% of men (versus 3.3% (*p* < 0.001) and 0.7% (*p* < 0.001) of women) (Table 1). Lack of fruits and vegetables consumption, lack of physical activity and weight excess were found in 36.9%, 60.7% and 65.4% of participants respectively.

**Table 1:** Prevalence of cardiovascular risk factors among participants according to the sex, the age group and the socioeconomic level.

Risk factors		Total	Sex		Age				Socioeconomic level			
		Total (n%)	Male (n%)	Female n(%)	*p*	≤ 40 years n(%)	> 40 years n(%)	*p*	Low n(%)	Medium n(%)	High n(%)	*p*
Lack of physical activity	Yes	1195(60.7)	433(56.0)	762(63.7)	0.001	618(60.2)	574(61.1)	0.687	84(58.7)	999(61.4)	109(55.6)	0.261
	No	775(39.3)	340(44.0)	435(36.3)		409(39.8)	366(38.9)		59(41.3)	628 (38.6)	87 (44.4)	
Lack of fruits and vegetables consumption	Yes	726(36.9)	296(38.2)	430(36.0)	0.317	435(42.4)	291(30.0)	< 0.001	65(45.5)	602(37.0)	56(28.7)	0.006
	No	1242(63.1)	478(61.8)	764(64.0)		592(57.6)	647(69.0)		78(54.5)	1024(63.0)	139(71.3)	
Current tobacco use	Yes	410(20.8)	370(47.7)	40(3.3)	< 0.001	219(21.3)	191(20.3)	0.574	39(27.3)	329 (20.1)	42 (21.5)	0.127
	No	1565(79.2)	406(52.3)	1159(96.7)		810(78.7)	752(79.7)		104(72.7)	1304(79.9)	153(78.5)	
Current alcohol consumption	Yes	124(6.4)	116(15.5)	8(0.7)	< 0.001	83(8.2)	41(4.4)	0.001	5(3.5)	104(6.5)	15(7.9)	0.261
	No	1815(93.6)	631(84.3)	1184(99.3)		925(91.8)	887(95.6)		137(96.5)	1500(93.5)	175(92.1)	
Weight status	Normal	679(34.7)	314(40.8)	365(30.7)	< 0.001	493(48.4)	185(19.8)	< 0.001	42(29.4)	562(34.8)	74(38.1)	0.146
	Overweight	659(33.7)	305(39.7)	354(29.8)		329(32.3)	329(35.1)		44(30.8)	546 (33.8)	68 (35.1)	
	Obesity	620(31.7)	150(19.5)	470(39.5)		197(19.3)	422(45.1)		57(39.9)	509 (31.5)	52 (26.8)	
Android obesity	Yes	924(47.1)	153(19.9)	771(64.6)	< 0.001	342(33.6)	580(61.5)	< 0.001	82(56.9)	762 (47.0)	77 (39.7)	0.007
	No	1039(52.9)	617(80.1)	422(35.4)		675(66.4)	363(38.5)		62(43.1)	859(53.0)	117(60.3)	
Hypercholesterolemia	Yes	107(5.4)	21(2.7)	86(7.2)	< 0.001	8(0.8)	99(10.5)	< 0.001	10 (6.9)	90 (5.5)	7 (3.6)	0.371
	No	1869(94.6)	756(97.3)	1113(92.8)		1021(99.2)	845(89.5)		134(93.1)	1542(94.5)	189(96.4)	
Diabetes	Yes	138(7.0)	44(5.7)	94(7.8)	0.064	11(1.1)	127(13.4)	< 0.001	9 (6.3)	119 (7.3)	10 (5.1)	0.491
	No	1838(93.0)	733(94.3)	1105(92.2)		1017(98.9)	818(86.6)		135(93.7)	1513(92.7)	186(94.9)	
High blood pressure	Yes	197(10.0)	50(6.4)	147(12.3)	< 0.001	18(1.7)	178(18.8)	< 0.001	19(13.2)	168 (10.3)	9 (4.6)	0.017
	No	1780(90.0)	727(93.6)	1053(87.7)		1011(98.3)	767(81.2)		125(86.8)	1465(89.7)	187(95.4)	

Lack of physical activity and weight excess were significantly more frequent in women than in men (Table 1). The same was observed with the history of hypercholesterolemia or hypertension, which were also significantly more frequent among women (Table 1). Among participants over 40 years, obesity, android obesity, hypercholesterolemia, diabetes or hypertension were significantly more frequent than among those under the age of 40 years (Table 1). Otherwise, lack of fruits and vegetables consumption, android obesity and history of hypertension were significantly more frequent among adults with low socioeconomic level compared to those with higher socioeconomic levels (Table 1). On the other hand, lack of physical activity was significantly more frequent among participants with weight excess (63.5% versus 55.3%, *p* < 0.001) and those with diabetes (69.6% versus 60%, *p* = 0.026) than among those having not these conditions (Table 2).

**Table 2:** Prevalence of cardiovascular risk factors among participants according to their health conditions.

		Overweight or obesity			Diabetes			High blood pressure			Cardiovascular disease		
		Yes n(%)	No n(%)	*p*	Yes n(%)	No n(%)	*p*	Yes n(%)	No n(%)	*p*	Yes n(%)	No n(%)	*p*
**Risk factors**													
Lack of physical activity	Yes	820 (63.5)	375 (55.3)	< 0.001	96 (69.6)	1098 (60.0)	0.026	129 (65.5)	1066 (60.1)	0.144	30(60.0)	1164 (60.7)	0.925
	No	472 (36.2)	303 (44.7)		42 (30.4)	733 (40.0)		68 (34.5)	707 (39.9)		20 (40.0)	755 (39.3)	
Lack of fruits and vegetables consumption	Yes	430 (33.3)	296 (43.8)	< 0.001	56 (40.6)	669 (36.6)	0.347	65 (33.0)	661 (37.3)	0.232	19 (36.5)	706 (36.9)	0.961
	No	862 (66.7)	380 (56.2)		82 (59.4)	1160 (63.4)		132 (67.0)	1110 (62.7)		33 (63.5)	1209 (63.1)	
Current tobacco use	Yes	220 (17.0)	190 (28.0)	< 0.001	14 (10.1)	396 (21.6)	0.001	17 (8.6)	393 (22.1)	< 0.001	8 (15.4)	401 (20.9)	0.336
	No	1077 (83.0)	488 (72.0)		124 (89.9)	1440 (78.4)		180 (91.4)	1385 (77.9)		44 (84.6)	1521 (79.1)	
Current alcohol consumption	Yes	58 (4.6)	66 (9.8)	< 0.001	3 (2.2)	121 (6.7)	0.038	7 (3.6)	117 (3.6)	0.091	1 (1.9)	123 (6.5)	0.181
	No	1210 (95.4)	605 (90.2)		133 (97.8)	1681 (93.3)		188 (96.4)	1627 (93.3)		51 (98.1)	1763 (93.5)	

When the investigators measured blood pressures, they highlighted that among the 197 participants who reported a history of hypertension, 143 (72%) had elevated blood pressures. On the other hand, among the 1776 subjects who were not followed for hypertension, 464 (26.2%) had elevated blood pressures.

Assessment of blood pressure, glycaemia, cholesterolemia and BMI were performed among 69.2%, 46%, 40.4%, and 36.6% of the 1246 participants who have visited a healthcare facility in the past year whereas the screening for tobacco use and for android obesity were reported by 13.5% and 5.7% of these latter (Table 3). All these practices were significantly more frequent in women, in participants above 40 years, those with weight excess, diabetes, hypertension or CVD except for tobacco use (Tables 3 and 4).

**Table 3:** Screening and health education practices related to cardiovascular risk factors among te participants that have visited a healthcare facility during the previous year, according to their sociodemographic characteristics.

			Sex		Age				Line of the visited healthcare facility				Sector of the healthcare sector			
		Total	Male	Female	p	≤ 40 years	> 40 years	p	1^st^	2^nd^	3^rd^	p	Public	Private	Public and Private	p
**Screening for**																
Tobacco use	Yes	168(13.5)	114(30.2)	54(6.3)	< 0.001	88(13.8)	80(13.4)	0.849	35(10.1)	31(9.6)	43(14.3)	0.045	47(14.1)	65(15.0)	16(14.5)	0.941
	No	1072(86.5)	264(69.8)	808(93.7)		552(86.3)	518(86.6)		311(89.9)	291(90.4)	257(85.7)		286(85.9)	368(85.0)	94(85.5)	
Obesity	Yes	455(36.6)	99(26.1)	356(41.3)	< 0.001	205(34.2)	249(38.9)	0.083	110(31.9)	110(34.3)	107(35.7)	0.419	106(31.9)	184(42.3)	44(40.4)	0.012
	No	787(63.4)	280(73.9)	507(58.7)		395(65.8)	391(61.1)		235(68.1)	211(65.7)	193(64.3)		226(68.1)	251(57.7)	65(59.6)	
Android obesity	Yes	70(5.7)	12(3.2)	58(6.8)	0.013	33(5.5)	37(5.9)	0.805	18(5.3)	22(6.9)	22(7.4)	0.344	17(5.2)	22(5.1)	9(8.3)	0.393
	No	1161(94.3)	363(96.8)	789(93.2)		564(94.5)	595(94.1)		324(94.7)	297(93.1)	274(92.6)		313(94.8)	409(94.9)	99(91.7)	
Hyperglycemia	Yes	570(46.0)	149(39.3)	421(48.9)	0.002	204(34.2)	365(56.9)	< 0.001	152(44.2)	155(48.1)	148(49.5)	0.206	147(44.4)	193(44.4)	49(45.4)	0.982
	No	670(54.0)	230(60.7)	440(51.1)		393(65.8)	276(43.1)		192(55.8)	167(51.9)	151(50.5)		184(55.6)	242(55.6)	59(54.6)	
Hypercholesterolemia	Yes	499(40.4)	134(35.5)	365(42.5)	0.022	157(26.3)	341(53.5)	< 0.001	136(39.7)	141(43.8)	125(41.9)	0.422	136(41.1)	166(38.4)	43(39.4)	0.757
	No	737(59.6)	243(64.5)	494(57.5)		440(73.7)	296(46.5)		207(60.3)	181(56.2)	173(58.1)		195(58.9)	266(61.6)	66(60.6)	
High blood pressure	Yes	860(69.2)	237(62.7)	623(72.0)	0.001	356(59.3)	503(78.5)	< 0.001	230(66.9)	230(71.4)	213(71.0)	0.234	224(67.5)	305(70.1)	84(77.1)	0.165
	No	383(30.8)	141(37.3)	242(28.0)		244(40.7)	138(21.5)		114(33.1)	92(28.6)	87(29.0)		108(32.5)	130(29.9)	25(22.9)	
**Health education about**																
The dietary habits	Yes	401(32.3)	101(26.6)	300(34.8)	0.004	128(21.4)	273(42.5)	< 0.001	117(33.8)	109(33.7)	93(31.0)	0.532	115(34.5)	151(34.7)	37(33.6)	0.978
	No	842(67.7)	279(73.4)	563(65.2)		471(78.6)	369(57.5)		229(66.2)	214(66.3)	207(69.0)		218(65.5)	284(65.3)	73(66.4)	
Physical activity	Yes	332(26.7)	94(24.7)	238(27.6)	0.292	91(15.2)	240(37.4)	< 0.001	93(26.9)	89(27.6)	74(24.7)	0.952	97(29.1)	128(29.4)	31(28.4)	0.979
	No	910(73.3)	286(75.3)	624(72.4)		507(84.8)	402(62.6)		253(73.1)	234(72.4)	225(75.3)		236(70.9)	307(70.6)	78(71.6)	
Tobacco cessation (for tobacco users)	Yes	74(37.4)	69(40.4)	5(18.5)	0.029	30(30.6)	44(44.0)	0.057	12(35.3)	14(29.2)	26(40.6)	0.009	22(39.3)	27(37.0)	9(50.0)	0.599
	No	124(62.6)	102(59.6)	22(81.5)		68(69.4)	56(56.0)		22(64.7)	34(70.8)	38(59.4)		34(60.7)	46(63.0)	9(50.0)	
Information about the overall cardiovascular risk	Yes	134(10.8)	57(15.2)	77(9.0)	0.001	46(7.7)	87(13.6)	0.001	37(10.7)	30(9.3)	32(10.7)	0.669	38(11.4)	51(11.9)	12(11.0)	0.964
	No	1102(89.2)	319(84.8)	783(91.0)		550(92.3)	551(86.4)		308(89.3)	293(90.7)	267(89.3)		294(88.6)	379(88.1)	97(89.0)

**Table 4:** Screening and health education practices related to cardiovascular risk factors among the participants that have visited a healthcare facility during the previous year, according to their health conditions.

		Overweight or obesity			Diabetes			High blood pressure			Cardiovascular disease		
		Yes n(%)	No n(%)	p	Yes n(%)	No n(%)	p	Yes n(%)	No n(%)	p	Yes n(%)	No n(%)	p
**Screening for**													
Tobacco use	Yes	107(12.5)	61(15.9)	0.102	22(18.3)	146(13.0)	0.108	24(13.6)	144(13.5)	0.971	8(17.4)	160(13.4)	0.438
	No	750(87.5)	322(84.1)		98(81.7)	973(87.0)		152(86.4)	920(86.5)		38(82.6)	1034(86.6)	
Obesity	Yes	340(39.6)	115(30.0)	0.001	85(69.7)	369(33.0)	< 0.001	98(55.1)	357(33.6)	< 0.001	16(34.8)	439(36.7)	0.791
	No	519(60.4)	268(70.0)		37(30.3)	750(67.0)		80(44.9)	707(66.4)		30(65.2)	757(63.3)	
Android obesity	Yes	59(6.9)	11(2.9)	0.005	14(11.8)	56(5.0)	0.003	17(9.8)	53(5.0)	0.012	-	70(5.9)	0.090
	No	791(93.1)	370(97.1)		105(88.2)	1055(95.0)		157(90.2)	1004(95.0)		46(100.0)	1115(94.1)	
Hyperglycemia	Yes	443(51.6)	127(33.2)	< 0.001	115(94.3)	455(40.7)	< 0.001	138(77.5)	432(40.7)	< 0.001	36(78.3)	534(44.7)	< 0.001
	No	415(48.4)	255(66.8)		7(5.7)	662(59.3)		40(22.5)	630(59.3)		10(21.7)	660(55.3)	
Hypercholesterolemia	Yes	398(46.5)	101(26.6)	< 0.001	110(90.9)	389(34.9)	< 0.001	130(73.4)	369(34.8)	< 0.001	34(73.9)	465(39.1)	< 0.001
	No	458(53.5)	279(73.4)		11(9.1)	725(65.1)		47(26.6)	690(65.2)		12(26.1)	725(60.9)	
High blood pressure	Yes	643(74.8)	217(56.7)	< 0.001	113(92.6)	746(66.6)	0.001	165(92.7)	695(65.3)	< 0.001	42(91.3)	818(68.3)	0.001
	No	217(25.2)	166(43.3)		9(7.4)	374(33.4)		13(7.3)	370(34.7)		4(8.7)	379(31.7)	
**Health education about**													
The dietary habits	Yes	326(37.9)	75(19.6)	< 0.001	100(82.0)	301(26.9)	< 0.001	125(70.2)	276(25.9)	< 0.001	24(52.2)	377(31.5)	0.003
	No	534(62.1)	308(80.4)		22(18.0)	819(73.3)		53(29.8)	789(74.1)		22(47.8)	820(68.5)	
Physical activity	yes	278(32.4)	54(14.1)	< 0.001	88(72.1)	244(21.8)	< 0.001	105(59.0)	227(21.3)	< 0.001	22(47.8)	310(25.9)	0.001
	No	581(67.6)	329(85.9)		34(27.9)	875(78.2)		73(41.0)	837(78.7)		24(52.2)	886(74.1)	
Tobacco cessation (for tobacco users)	Yes	41(42.3)	31(35.2)	0.327	8(72.3)	64(36.8)	0.018	9(60.0)	63(37.1)	0.081	5(71.4)	67(37.6)	0.072
	No	56(57.7)	57(64.8)		3(27.3)	110(63.2)		6(40.0)	107(62.9)		2(28.6)	111(62.4)	
Information about the overall cardiovascular risk	Yes	103(12.0)	31(8.2)	0.043	28(23.3)	106(9.5)	< 0.001	35(19.9)	99(9.3)	< 0.001	7(15.2)	127(10.7)	0.331
	No	753(88.0)	349(91.8)		92(76.7)	1009(90.5)		141(80.1)	961(90.7)		39(84.8)	1063(89.3)	

Health education practices related to physical activity and dietary habits were reported by 26.7% and 32.3% of those who have visited a healthcare facility in the past year. These preventive services were significantly more frequently reported by those over 40 years and those with weight excess, diabetes, hypertension or CVD (Tables 3 and 4) but did not vary with healthcare lines or sectors, while advices to quiet tobacco were reported by 37.4% of tobacco users that have visited a healthcare facility in the past year (Table 3).

Information about its overall CVD risk was reported by 10.8% of participants. This practice was significantly more frequently reported by male participants (15.2%), those above 40 years (13.6%) (Table 1), those with weight excess (12%), those with diabetes (23.3%) and those with hypertension (19.9%) (Table 3).

## Discussion

The current study is between the rare studies led in Tunisia to assess the screening and health education practices related to the CVD RFs among adults. The current results would guide the implementation of future prevention actions to reduce the burden of CVD mortality in Tunisia.

Results of the present study revealed that despite the high frequency of several CVD RFs among the adults of Sousse, health professionals are still more oriented toward secondary prevention services than toward primary prevention services. Indeed, screening for CVD RFs and health education practices were rare in the visited healthcare facilities of Sousse especially those related to behavioral RFs among adults under the age of 40 years or without chronic condition like obesity, diabetes, hypertension or CVD.

Smoking and alcohol consumption were reported by 47.7% and 15.5% of male participants. These results are in line with those of the national study "Tunisia Health Survey" (THES) led in 2016 which have shown similar prevalences of 48.3% and 11.7% in males^21^. Although tobacco use was frequent among males, screening for this risk behavior was reported by only 30.2% of them. Nevertheless, a meeting with a health professional represents an opportunity for a minimal advice to quiet tobacco. In European countries, screening for tobacco use is much more common^22^. The action plans introduced since 1987 for a tobacco-free Europe represent models to be followed in Tunisia^22^.

Lack of physical activity was another highly prevalent risk behavior among participants (60.7%). On the other hand, advices from health professionals to be more active were reported by only 26.7% of participants that have visited a healthcare facility in the past year. These results are similar to those observed during the EUROASPIRE III study conducted in 12 European countries where sedentary habits were reported by a majority of participants with only 1/10 receiving specific advice from a health professional^22^. In participants with history of diabetes or hypertension, screening for this risk behavior was more frequently reported (by 72.1% and 59% respectively). However, in participants with weight excess, the frequency of this practice was of 34.4%. This finding suggests that Tunisian healthcare providers are much more oriented towards secondary prevention than towards primary prevention.

Regarding dietary habits, 36.9% of participants were not consuming the recommended amount of fruits and vegetables whereas advises to eat more fruits and vegetables were reported by only 32.3% of the participants that they have visited a healthcare facility in the past year. This also joins the results of the EUROASPIRE III study^22^. On another note, contrary to participants with weight excess which reported similar frequency of dietary education, participants with diabetes, hypertension or CVD reported more frequently dietary education. This reflects once again the orientation towards treatment rather than towards prevention in terms of CVD control in Tunisia.

Concerning weight excess, overweight, obesity, and android obesity were found in 33.7%, 31.7%, and 47.1% of participants respectively. The current obesity prevalence is not far from the national prevalences of 2016 reported by the WHO (27%) and the THES (26.2%)^21,23^. Among participants who have visited healthcare facilities during the previous year, 36.6% reported weight measurement and only 5.7% reported waist measurement. These low frequencies of assessment were reported across the different lines of care and across the two health sectors. Even in overweight participants, only 39.6% and 6.9% of participants reported respectively these measurements while they represent simple, rapid and inexpensive measures that are essential for health education.

In regards to blood pressures, history of hypertension was reported by 10% of participants. However, investigators found that 72% of them had elevated blood pressures. This proportion exceeds that of 43% revealed by the EUROASPIRE IV study conducted between 2012 and 2014 in 26 European countries^24^. Indeed, it is estimated that the control of hypertension is four times lower in developing countries compared to developed countries^25,26^. Furthermore, 26.2% of participants without history of hypertension had also elevated blood pressures. In fact, the national prevalence of hypertension was of 28.7% in 2016^21^. In line with these results, the ETHNA-Tunisia study conducted in the first line among 5802 participants highlighted a hypertension prevalence of 26.9% in men and 28.4% in women^27^. Otherwise, assessment of blood pressures in the visited healthcare facilities was the highest in patients with hypertension or diabetes. Actually, this activity is included in the national program for the management of diabetes and hypertension.

With regards to diabetes, it was reported by 7% of participants. Nonetheless, the national prevalence of diabetes was of 15.5% in 2016^21^. The ETHNA-Tunisia study showed a higher prevalence of 19.2%^27^. In the Eastern Mediterranean region, similar prevalences were stated in 2016 (16.2% in Egypt, 13.7% in Libya, and 12.4% in Morocco)^28^. Lack of diabetes screening practices could explain an eventual underestimation of diabetes prevalence among participants. In fact, among those with weight excess for example, the frequency of this practice was 74.8%.

Similarly, hypercholesterolemia was reported by 5.4% of participants while the national prevalence of this metabolic problem was 8.4% in 2009^14^. This prevalence among participants is also lower than that recently recorded in the Eastern Mediterranean countries^28^. The low frequency of cholesterolemia assessment (40.4%) supports the hypothesis of an under diagnosis of dyslipidemia among the adults of Sousse.

Finally, information about its overall CVD risk was reported by only 10.8% of participants. The use of a cardiovascular risk prediction tool would facilitate this practice and guide health education activities in the Tunisian healthcare services^29,30^.

Between 2013 and 2020, in parallel with the national strategy for the management of hypertension and diabetes and that implemented to reduce tobacco use, two supplementary strategies were developed in Tunisia to promote healthy lifestyle. The first strategy aimed at reducing obesity and focused on multisectoral actions mainly in food industry such as salt and fat reduction and products labelling. The second strategy aimed at reducing the burden of cancers with emphasis on tobacco use reduction, promoting physical activity and healthy nutrition. These strategies share several objectives. However, financial difficulties did not allow implementing properly these strategies. Accordingly, it was proposed to combine them in only one multisectoral strategy to fight against NCDs. The political instability should no longer delay the implementation of this comprehensive strategy in Tunisia especially that a national guide of health education was elaborated in addition to a communication plan, a community participation plan and a framework for multisectoral regional plans related to NCDs prevention^13^. Multisectoral committees are required to coordinate the related prevention activities at the regional and national levels. Besides, the training of health professionals in terms of primary prevention of NCDs is necessary in order to provide affordable prevention services like the screening for CVD RFs and health education in the healthcare facilities. International experiences have provided successful models of health education implementation based on multidisciplinary teams with the involvement of the patients and their family members. This is the case of the Family Medicine Centers in Canada and Health Houses in France where team-based and patient-centered care services are offered^31^.

The results of the present study should be interpreted with taking into account some considerations related to the study method. Firstly, because of the cross-sectional nature of the study, it was possible to report statistical associations but not causal relationships. It is also important to consider that to reduce both: the geographic dispersion of the sample and the cost of the survey, a two-stages cluster sampling was performed. This sampling method remain a random approach. Besides, participants accounted for 1977. These elements are in favor of the sample representativity and would result in generalizable results. Furthermore, practices of healthcare professionals in the healthcare facilities follow national programs that are similarly operationalized in the different regions of the country. Otherwise, habits, medical history, screening and health education practices in the healthcare facilities were reported by the participants retrospectively. This could lead to a social desirability bias or a recall bias. As a result, the estimated prevalences may be underestimated or overestimated. Nonetheless, the investigators explained the aims of the study, anonymity and the confidential data processing prior to data collection. This would have reduced this potential information bias. Finally, 20.3% of the participants did not have social security coverage. This would have minimized opportunities for screening and health education for them and may have caused underestimation of their practices. To avoid this risk, screening and health education practices were reported only among those who have visited a healthcare facility in the past year, i.e. 63% (1246) of the participants.

## Conclusion

CVD RFs, especially behavioral ones, are highly prevalent among the adults of Sousse. Their management in the healthcare facilities is much more oriented towards the secondary prevention than towards primary prevention. It is time to operationalize a national multisectoral health promotion program in order to reduce the burden of CVD mortality in Tunisia.

## Data Availability

The datasets analyzed during the current study are not publicly available due to limitations of ethical approval involving the patient data and anonymity but are available from the corresponding author on reasonable request.
